# Database and registry research in pediatric anesthesiology

**DOI:** 10.1097/ACO.0000000000001502

**Published:** 2025-04-28

**Authors:** Joseph Cravero, Morgan L. Brown

**Affiliations:** Department of Anesthesiology, Critical Care, and Pain Medicine Boston Children’s Hospital, Boston, Massachusetts, USA

**Keywords:** anesthesiology, data registry, database, outcomes research, pediatric

## Abstract

**Purpose of the review:**

The collection and analysis of large amounts of data has revolutionized almost every aspect of our lives. In this review, we will explore several of the databases that are providing data on pediatric anesthesiology practice and the recent reports that have been published.

**Recent findings:**

Studies from various multicenter databases provide information on outcomes from multiple aspects of pediatric anesthesia care provision. Large databases or registries include detailed information on individual anesthetic practice, airway management, regional anesthetic practice, adverse events, and cardiac anesthesia. These collaboratives are also providing information on health systems and benchmarking of effectiveness and efficiency of care.

**Summary:**

For medical professionals, the ability to collect and learn from large datasets is not new but continues to evolve and improve as technology improves and the science of data analytics has been revolutionized. While the detail and accuracy of observational large data collaboratives may be limited, their ability to describe practice patterns, provide benchmarking for performance, and analyze outcome parameters has the potential to revolutionizing the practice of anesthesia in the future.

**KEY POINTS**

- The Multicenter Perioperative Outcomes Group is a large data collaborative spanning the breadth of the field of anesthesiology with a pediatric component that has investigated multiple outcomes related to pediatric anesthesia, most recently finding that hypoxia during one-lung ventilation is related to patient demographic factors, but has no relationship to technique used for one-lung ventilation.
- Analysis of pediatric cardiac anesthesia outcomes from the Society of Thoracic Surgeons’ Congenital Heart Surgery & Congenital Cardiac Anesthesia Society database showed that increasing postoperative hematocrit above 42% was associated with increasing operative mortality and major complications. Recently, this database has also been used to look at anesthesia ready time and its predictors.
- The Pediatric Difficult Intubation Registry recently reported a study that found standard videolaryngoscopy blades were more successful for the intubation of children weighing less than 5 kg, as well as other manuscripts on the management of difficult airways.

## INTRODUCTION

Evidence of the revolution in data acquisition and analysis is everywhere. Opening an internet browser reveals customized advertisements that have been chosen based on your previous browsing history or analysis of purchases by individuals who have purchased similar products. Data collection and analysis (utilizing artificial intelligence) will dominate our future with technologies like ChatGPT or self-driving vehicles roaming major cities.

Randomized, controlled, trials (RCTs) continue to represent the gold standard for linking outcomes with care parameters given the ability of this design to minimize confounding. RCTs are expensive to perform and in pediatrics, it can be challenging to receive Institutional Review Board approval given ethical concerns. In addition, many aspects of pediatric anesthesia are changing so rapidly that by the time an RCT can be conceived, funded, approved, and completed, some of the data is irrelevant due to changes in the nature of the way the specialty is practiced. Thus, while RCTs remain the gold standard, other study designs are necessary.

Observational databases are crucial for managing patient information, tracking outcomes, performing quality improvement, improving education, and conducting research in pediatric anesthesia. There is evidence that well-conducted large-data observational trials provide similar findings when compared to controlled trials that investigate the same outcomes [1,2]. This review will highlight recent publications in the field of pediatric anesthesia that have been the product of observational database analyses. Table 1 details these and other available databases in pediatric anesthesia.

**Table 1.** Available commonly used databases and registries for with pediatric anesthesia data

Name of database or registry	Acronym	Types of patients or cases in the database
Multicenter Perioperative Outcomes Group	MPOG	Preoperative, intraoperative, and early postoperative data
Pediatric Anesthesia COVID Collaborative	PEACOC	COVID operative cases
Pediatric Difficult Intubation Registry	PeDiR	Difficult or anticipated difficult airways
Pediatric Craniofacial Collaborative Group	PCCG	Craniofacial surgical cases
Pediatric Regional Anesthesia Network	PRAN	Regional anesthesia use
Society for Pediatric Anesthesia Improvement Network	SPAIN	Perioperative pain in surgical procedures
Society of Thoracic Surgeons Congenital Cardiac Anesthesia Society	STS-CCAS	Cardiac surgical cases
Wake Up Safe	WUS	Adverse events intraoperatively and early postoperatively

## MULTICENTER PERIOPERATIVE OUTCOMES GROUP

The Multicenter Perioperative Outcomes Group (MPOG) is a large data collaborative spanning the breadth of the field of anesthesiology. The group was founded in 2008 and has a state mission to use data to understand outcomes and develop policies, procedures, and the infrastructure required for collaborative outcomes research in anesthesiology. MPOG has approximately 60 participating medical centers in the USA and Europe. Over its existence, MPOG has accumulated millions of patient encounters.

MPOG differs from other observational databases, in that it extracts the majority of an anesthetic record and combines this with preoperative and some postoperative data. This is all done through electronic data transfer without the use of data abstractors.

MPOG has a separate pediatric subgroup that collects and analyzes data on pediatric anesthesia and perioperative care. This database has been used to examine the practice of one-lung ventilation in school-age children [3^▪▪^]. This study evaluated children between 4 and 17 years of age undergoing one-lung ventilation. Hypoxemia was defined as oxygen saturation less than 90% for 3 min or longer, and severe hypoxemia was defined as the same threshold for 5 min or longer. The factors that were considered included age, weight, American Society of Anaesthesia - Physical Status (ASA-PS) of III or greater, duration of one-lung ventilation, preoperative oxygen saturation, technique for one-lung ventilation, operative side, video-assisted thoracoscopic surgery, tidal volume strategy, PEEP strategy, and procedure type.

In the younger (4 to 9 years) and older (10 to 17 years) cohorts, the rates of hypoxia were 10.5% and 7.5%, respectively. Severe hypoxia was also more common in the younger patients at 6.1% vs. 4.2%. An initial oxygen sat of less than 98% was found to be an independent predictor of hypoxemia in both age cohorts. No other factors were found to be specifically related to hypoxia in the younger cohort. For the 10 to 17 years aged group, extremes of weight, and right-sided cases were associated with an increased risk of hypoxemia. Surprisingly, there was no correlation with the method used for lung isolation of the site involved in the data collection and the hypoxemia rate. The authors conclude that the rate of hypoxemia in school-age children during one-lung ventilation appears to be higher than that seen in adult patients. These findings are important for anesthesia planning in children requiting single-lung ventilation. The combined experience of multiple centers is necessary for a study on such an infrequent procedure.

Another study from the MPOG pediatric group evaluated risk factors for intraoperative hypoglycemia in children [4]. In this study, the authors described a cohort of patients who experienced intraoperative hypoglycemia and determined the factors associated with this finding from 12 institutions participating in MPOG. They defined hypoglycemia as a glucose less than 60 mg/dl. Glucose data was derived from 26 142 out of 394 231 cases. Hypoglycemia was found in 3.9% of patients. After multivariable logistic regression age <30 days, age from 30 days to 5 years, weight <5%, ASA-PS status >/= III, presence of a gastric or jejunal tube, history of poor feeding, and abdominal surgery were all identified as independent risk factors. Given the risk of neurologic injury with hypoglycemia, the authors propose that patients at high risk for hypoglycemia be considered for improved blood glucose monitoring.

## THE PEDIATRIC DIFFICULT INTUBATION REGISTRY

The Pediatric Difficult Intubation Registry (PeDiR) is a registry collaborative dedicated to assessing, understanding, and improving the outcomes of children who are known to have difficult direct laryngoscopy. The group aims to collect data to provide evidence for quality improvement, and research. This database collects information on patients who were identified by a pediatric anesthesiologist as either a difficult intubation or an anticipated difficult intubation who report details regarding airway management strategies.

The collaborative has successfully published multiple papers concerning difficult airway management. In 2021 Peyton *et al*. [5] authored a report that compared videolaryngoscopy using standard blades vs. nonstandard blades (hyperangulated) in patients whose data were in the PeDiR. The authors compared success rates for initial and subsequent attempts at intubation and also evaluated technical difficulties.

Standard blades were found to be significantly more successful in children weighing less than 5 kg on the first attempt at intubation (51% vs. 26%) and eventual intubation (81% vs. 58%). In multivariable analysis, standard blades were found to have a three times greater odds of success at initial tracheal intubations compared with nonstandard blades (odds ratio 3.0) whereas for subsequent attempts the improvement was 2.6 times. In contrast to these findings, there was no difference in success rates between videolaryngoscope blade types in patients larger than 5 kg. In their conclusions, they propose that using a standard blade during videolaryngoscopy is a logical choice for patients under 5 kg.

Another recent report evaluated the use of neuromuscular blocking agents and ventilation techniques on complications in the PeDiR [6]. This particular analysis evaluated patients who were intubated during spontaneous vs. controlled ventilation after the administration of neuromuscular blocking agents.

Propensity score matching was used to match the nonairway-related characteristics of the cohorts. The results were collected from 1289 anticipated difficult intubations. Of these, 39% were managed with spontaneous ventilation while 35% were managed with controlled ventilation after neuromuscular blocking agents and 26% with controlled ventilation without a neuromuscular blocker. Complications occurred in 18.8% with 1.9% categorized as severe. The spontaneous ventilation cohort had a higher rate of nonsevere complications when compared to the controlled ventilation group (OR 2.07). Regression analysis after propensity matching confirmed the increased rate of adverse events in spontaneously ventilated patients. The authors speculated that the difference could potentially be due to inadequate anesthetic depth and suggest that controlled ventilation is associated with fewer minor adverse events.

## PEDIATRIC ANESTHESIA COVID COLLABORATION

The Pediatric Anesthesia COVID Collaborative is a collaborative with the aim of evaluating the COVID-19 prevalence, institutional practices, and outcomes in pediatric surgical patients during the pandemic. In 2023 Kato *et al*. [7] reported on the prevalence of COVID among children undergoing anesthesia and surgery and assessed the independent risk factors for infection. Data was collected from 33 320 anesthesia encounters and included 265 children with COVID-19 infection. Infection rates decreased over time (*P* < 0.001). Rates of COVID-19 in pediatric patients presenting for surgery and anesthesia were consistently lower than in the general population. Independent risk factors of a positive COVID test for children were identified, including race, high ASA status, emergency cases as well as orthopedic and abdominal surgical interventions.

Another study from this collaborative surveyed the impact of COVID-19 on the practice of pediatric anesthesia including five major domains: equipment/medication, vaccination/testing, staffing, burnout, and economic repercussions [8]. The results of this broad-based survey of caregivers found that there were significant shortages of equipment and staff. Most indicated that burnout was a significant problem and noted that a large proportion of anesthesiologists considering retirement or decreasing their work hours. Suggestions for improving the workforce issues included increases in pay and work-hour flexibility.

There were major discrepancies between what anesthesiologists believe is important for job satisfaction and faculty retention compared to the initiatives that were implemented during the study period. These survey data may provide some insight for departmental and institutional leadership who are addressing these challenges.

## PEDIATRIC REGIONAL ANESTHESIA NETWORK

Pediatric Regional Anesthesia Network (PRAN) was formed as a data collaborative studying the practice, risks, and complications of pediatric regional anesthesia. PRAN has a data repository for benchmarking, research, and quality improvement as well as a framework for organizing future large-scale multicenter studies.

This organization has published multiple studies of their database, the latest of which was focused on the variability in the dosing of regional analgesia [9]. The primary aim of this study from Taenzer *et al.* was to determine the factors associated with dosing for the 10 most commonly performed PNBs in children. Data was collected for over 34 000 blocks. The authors concluded that significant variation in dosing was present among all age groups and all block types. Of all the factors in the database, the most important driver of relative dosing was the institution where the block was performed, indicating dosing may be more based on local culture than on evidence. The authors advocate for more data-driven and consistent practice and the need for more exacting studies to delineate dose requirements given the risk profile of local anesthetics.

## WAKE UP SAFE

Wake up Safe (WUS) is a national collaborative that collects data on perioperative anesthesia unexpected events to improve outcomes for children receiving anesthetic care as well as to educate members in improvement science. Cases with a reported adverse event have case details collected by the anesthesiology team including local root-cause-analyses.

In a report from 24 pediatric tertiary care hospitals participating in the WUS registry, this collaborative documented the incidence and causes of adverse events in diagnostic radiological studies requiring anesthesia [10]. In total, 175 486 anesthetics were included from 24 institutions across the USA. The overall incidence of adverse events was 0.05% which were categorized into serious and less serious events. Adverse events were more likely among patients with ASA status IV compared to ASA I patients (adjusted odds ratio, 8.9). Adverse events were generally rare, although the majority of AEs were related to anesthesia care.

Another recent study from this collaborative included a report on adverse events associated with deep extubation which found a small number of serious respiratory events, but none associated with long-term sequalae [11]. In addition, Wakimoto *et al.* [12] used WUS from this group to describe the nature and incidence of anaphylaxis during the perioperative time frame. Perioperative anaphylactic reactions occurred in 1:36 479 (0.003%). Antibiotics, neuromuscular blocking agents, and opioid analgesics were the main triggers. Finally, a report by Raghavan *et al.* [13] reviewed WUS data on perianesthetic neurological complications in children. The investigators found that the odds of a neurologic AE were significantly higher in children who were older or had ASA status of III or higher. Anesthesia contributed to 24 (18.9%) events and 37 (29.1%) events were preventable, including two deaths. Preventable factors broadly included inadequate preoptimization, complications during airway management and central venous catheter placement, and suboptimal patient positioning.

## CONGENITAL CARDIAC ANESTHESIA SOCIETY- SOCIETY OF THORACIC SURGEONS CONGENITAL HEART SURGERY DATABASE

A very helpful paper reviewing the use of databases in pediatric cardiac anesthesia and surgery was prepared by Vener *et al.* [14]. These authors describe over a dozen databases aggregating data on congenital heart disease. Most notably for pediatric anesthesiologists, the Congenital Cardiac Anesthesia Society (CCAS) has created a module within the Society of Thoracic Surgeons’ Congenital Heart Surgery Database (STS CHSD). This allows the anesthetic data to be combined with data that is meticulously collected by the surgical data coordinators including preoperative, operative, and postoperative outcomes. It also has multiple audits to ensure data quality is maintained.

In 2023 Brown used the data from the CCAS-STS CHSD to report on a care process issue of anesthesia ready time [15^▪▪^]. In this study, 44 418 cases were evaluated. They found a median time to readiness was 51 min and a longer time was found to be associated with lower weight, prematurity, chromosomal abnormalities, increasing medical comorbidities, involvement of a resident trainee, and the placement of a central line. Decreased time to readiness was associated with emergency cases, afternoon cases, and in situ invasive lines or an airway. This study is a nice example of the use of databases to measure and evaluate healthcare delivery systems and develop care performance metrics.

Another recent study of cardiac patients comparing outcomes with respect to transfusion practice was published by Long *et al.* [16]. The authors used multivariate logistical regression analysis to assess the relationship between postoperative hematocrit and operative mortality or major adverse outcomes. A total of 27 462 cases were included. For both types of cases, an increase in 5% increments in postoperative hematocrit above 42% was associated with an increase in operative mortality and major complications. These data demonstrate multicenter data are helpful to gain insights into transfusion practices.

## CONCLUSION

As with other areas of our lives, large data acquisition and analysis is now taking a large role in helping us understand the nature and directionality of pediatric anesthesia practice. The data from various collaboratives has helped to define the nature of outcomes in various areas of practice. The combining of data allows us to see the impact of practice across a large number of providers practicing in various settings. Furthermore, analysis of specific aspects of care has allowed for enhanced understanding of the workflows involved in pediatric anesthesia and factors that impact effectiveness and efficiency.

Improved integration of electronic medical records between institutions, and the leveraging of machine learning statistical analysis will doubtless increase the importance of large data analysis in our practice moving forward.

## Acknowledgements

*None.*

## Financial support and sponsorship

*None.*

## Conflicts of interest

*There are no conflicts of interest.*

## REFERENCES AND RECOMMENDED READING

Papers of particular interest, published within the annual period of review, have been highlighted as:

▪ of special interest
▪▪ of outstanding interest
